# Biodegradation Mechanisms of Patulin in *Candida guilliermondii*: An iTRAQ-Based Proteomic Analysis

**DOI:** 10.3390/toxins9020048

**Published:** 2017-02-08

**Authors:** Yong Chen, Huai-Min Peng, Xiao Wang, Bo-Qiang Li, Man-Yuan Long, Shi-Ping Tian

**Affiliations:** 1 Key Laboratory of Plant Resources, Institute of Botany, Chinese Academy of Sciences, Beijing 100093, China; cy123898@163.com (Y.C.); huaimin1216@163.com (H.-M.P.); wangxiao2@ibcas.ac.cn (X.W.); tsp@ibcas.ac.cn (S.-P.T.); 2 University of Chinese Academy of Sciences, Beijing 100049, China; 3 Department of Ecology and Evolution, The University of Chicago, Chicago, IL 60637, USA; mlong@uchicago.edu

**Keywords:** contamination, detoxification, mycotoxins, patulin, *Penicillium*, yeast

## Abstract

Patulin, a potent mycotoxin, contaminates fruits and derived products worldwide, and is a serious health concern. Several yeast strains have shown the ability to effectively degrade patulin. However, the mechanisms of its biodegradation still remain unclear at this time. In the present study, biodegradation and involved mechanisms of patulin by an antagonistic yeast *Candida guilliermondii* were investigated. The results indicated that *C. guilliermondii* was capable of not only multiplying to a high population in medium containing patulin, but also effectively reducing patulin content in culture medium. Degradation of patulin by *C. guilliermondii* was dependent on the yeast cell viability, and mainly occurred inside cells. E-ascladiol was the main degradation product of patulin. An iTRAQ-based proteomic analysis revealed that the responses of *C. guilliermondii* to patulin were complex. A total of 30 differential proteins involved in 10 biological processes were identified, and more than two-thirds of the differential proteins were down-accumulated. Notably, a short-chain dehydrogenase (gi|190348612) was markedly induced by patulin at both the protein and mRNA levels. Our findings will provide a foundation to help enable the commercial development of an enzyme formulation for the detoxification of patulin in fruit-derived products.

## 1. Introduction

Contamination of food and feed by fungal secondary metabolites, known as mycotoxins, is a global health issue [1]. Patulin is one of the important mycotoxins, and is produced by several species belonging to *Penicillium*, *Aspergillus*, *Paecilomyces, Byssochlamys*, and *Scopulariopsis* [2,3]. Among them, *Penicillium expansum* infects a wide range of fruits and vegetables and is the main producer of patulin [4]. Recently, the gene cluster involved in patulin biosynthesis pathway has been reported in *P. expansum* [5,6,7]. Since patulin is known to induce immunological, neurological and gastrointestinal diseases, long-term exposure to patulin may cause serious health issues in humans because [8,9].

The acceptable level of patulin in food has been limited in many countries worldwide. The European Commission [10] has set maximum permitted levels in apple juices (50 μg/kg), solid apple products (25 μg/kg) and, above all, fruity baby foods (10 μg/kg). Various strategies have been developed for patulin removal, including physical adsorption, chemical decontamination, and electromagnetic irradiation. However, several complications still remained in the application of available physical and chemical methods for the removal of patulin, such as safety issues, possible losses in the nutritional quality, limited efficacy and high costs [11]. As an alternative strategy, microbial degradation is considered as a potentially powerful method as compared to previously used approaches [12].

Many species of bacteria and fungi have the ability to degrade mycotoxins, including aflatoxin, fumonisin, ochratoxin-A and patulin [13,14]. It has been reported that patulin could be degraded by various yeast and bacteria strains [15,16]. Interestingly, some antagonistic yeast strains were also used to detoxify patulin, in addition to preventing infection of fungal pathogens on harvested fruits. For example, *Rhodosporidium kratochvilovae* LS11 and *Pichia caribbica* could degrade a high concentration of patulin both in vitro and in *P. expansum*-infected apple fruits [17,18]. Furthermore, some biodegradation products by those antagonistic yeasts have also been identified. For example, the major patulin degradation product formed in vitro by *R. kratochvilovae* LS11 was desoxypatulinic acid (DPA), which was reported to be nontoxic to different microorganisms [19]. However, at the present time, the mechanisms of biodegradation of patulin within yeast cells are still poorly understood.

*Candida guilliermondii* (teleomorph *Meyerozyma guilliermondii*) is an ascomycetous yeast that is widely distributed in the natural environment [20]. Due to its potential for biological control and biotechnological applications, this particular yeast has been intensively studied over the past 40 years [20]. As an antagonistic yeast, *C. guilliermondii* exhibited good biocontrol efficacy on various postharvest diseases [21,22,23,24]. The aim of the present study was (1) to investigate the degradation effect of *C. guilliermondii* on patulin; (2) to determine the major biodegradation products of patulin by *C. guilliermondii*; and (3) to explore the possible degradation mechanisms of patulin by *C. guilliermondii*.

## 2. Results

### 2.1. Biodegradation of Patulin by *C. guilliermondii*

**Figure 1:**
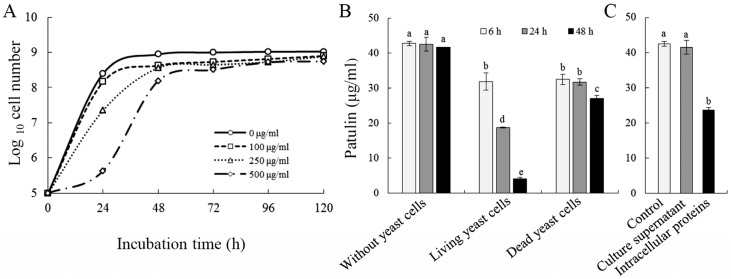
Biodegradation of patulin by *C. guilliermondii*. (**A**) Effects of patulin on the growth of *C. guilliermondii*; (**B**) Biodegradation assay of patulin by co-incubating with living and dead yeast cells; (**C**) Biodegradation assay of patulin by co-incubating with supernatant of the yeast culture and intracellular protein extracts. Error bars indicate standard deviations of the means from three replicates. Values followed by different letters are significantly different according to a Duncan’s multiple range test (*p* < 0.05).

The growth curve of *C. guilliermondii* in nutrient yeast dextrose broth (NYDB) medium with or without patulin was displayed in Figure 1A. At a concentration of 100 μg/mL, patulin slightly inhibited the growth of *C. guilliermondii*; this inhibitory effect improved as the concentration of patulin increased in the culture medium. A high concentration of patulin (500 μg/mL) markedly decreased the multiplication rate of the yeast, especially during the first 48 h of yeast culture growth. However, the population of yeast cells still reached a level of approximately 8 × 10^8^ cells/mL after an incubation period of 120 h.

The biodegradation effect of *C. guilliermondii* against patulin was determined by co-incubating patulin with yeast cells. During the incubation period, patulin content gradually decreased when living yeast cells were inoculated (Figure 1B). After 48 h, the concentration of patulin was reduced from 50 μg/mL to 4 μg/mL. When patulin was co-incubated with heat-killed dead cells, a slight decrease in patulin concentration was also observed. To further determine the localization of patulin biodegradation (intra- or extracelluar), patulin was co-incubated with supernatant of yeast culture or intracellular protein extract. As shown in Figure 1C, supernatant of the yeast culture did not reduce patulin concentration after 24 h of incubation. On the other hand, patulin was reduced by approximately 50% when patulin was co-incubated with intracellular protein extracts.

### 2.2. Biodegradation Products of Patulin by *C. guilliermondii*

**Figure 2:**
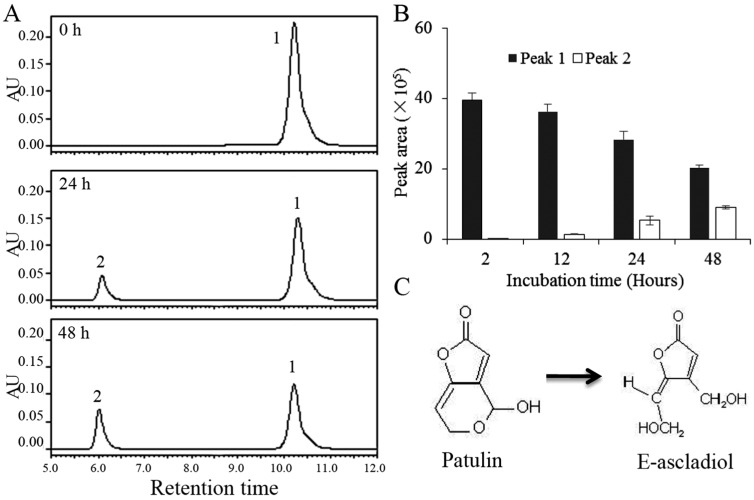
Biodegradation product of patulin by *C. guilliermondii*. (**A**) HPLC profiles of filtered mixtures before and after co-incubation of *C. guilliermondii* and patulin; (**B**) Changes in the peak area of peak 1 and peak 2 during the co-incubation; Error bars indicate standard deviations of the means from three replicates; (**C**) Conversion from patulin to E-ascladiol.

A biodegradation assay was performed by co-incubating patulin with living yeast cells, and HPLC profiles were examined to identify major breakdown products of patulin by *C. guilliermondii*. At 0 h, only a patulin peak with retention time of 10.2 min (peak 1) was detected (Figure 2A). After 24 h of co-incubation, a new peak with retention time of 6.0 min (peak 2) appeared, along with a decrease in the peak value of patulin. The retention time of the new product was consistent with an E-ascladiol standard. As the time of incubation increased, the area of peak 1 (patulin) decreased. Conversely, the area of peak 2 exhibited a gradual increase (Figure 2B). Meanwhile, no additional peaks appeared after patulin within the retention time of 20 min (Data not shown). Collectively, these results indicated that E-ascladiol is likely the main degradation product of patulin by *C. guilliermondii* (Figure 2C).

**Figure 3:**
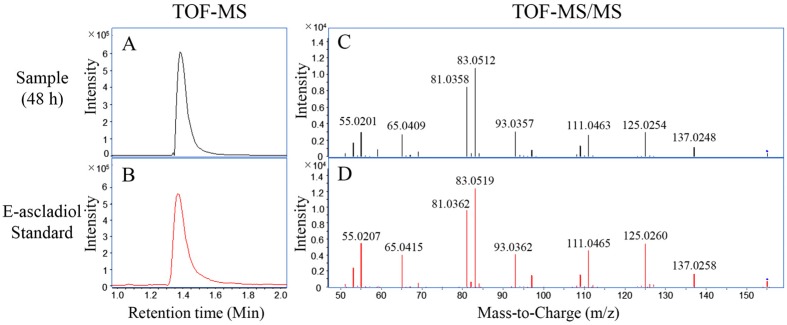
LC-TOF-MS and MS/MS analysis on the biodegradation product of patulin. (**A**,**B**) Extracted ion chromatograms of the 48 h sample (at *m*/*z* 155.035) and E-ascladiol standard (at *m*/*z* 155.0360); (**C**,**D**) Corresponding MS/MS profiles of the 48 h sample and E-ascladiol standard.

In order to further confirm the major degradation product, LC-TOF-MS and MS/MS analyses were performed for the E-ascladiol standard and a degradation sample from the 48 h time point. The peaks of E-ascladiol in the sample and standard were associated with the ion *m*/*z* 155.0358 and 155.0360 in the negative ionisation mode, respectively, and were identified as the [M − H]^−^ precursor. In comparison to the theoretical value (155.0350), errors of experimental *m*/*z* were below 2 ppm, which supported the assignment of the formulae C_7_H_8_O_4_. Profiles in Figure 3A,B show the extracted ion chromatogram (EIC) for these experimental *m*/*z* values. Signals in both the sample and standard were detected at a retention time of 1.4 min. Moreover, their MS/MS profiles were well matched as shown in Figure 3C,D. Collectively, these results confirmed that E-ascladiol was the main degradation product of patulin by *C. guilliermondii*.

### 2.3. Proteomic Responses of C. guilliermondii to Patulin

**Figure 4:**
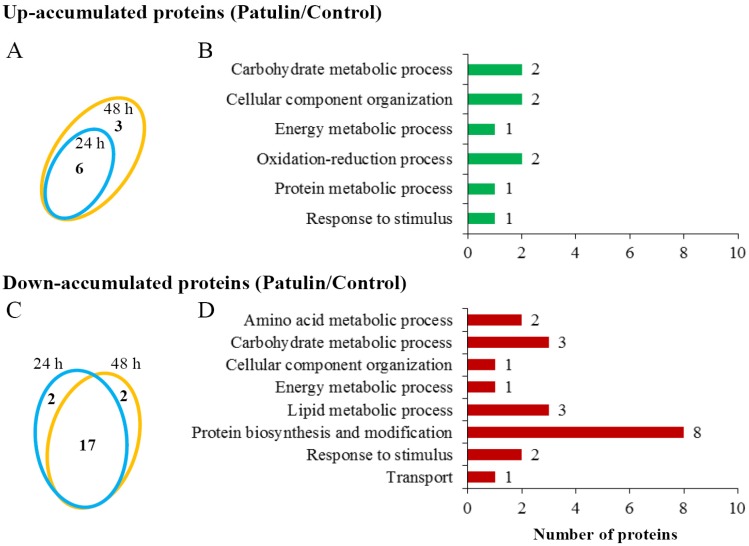
Differential proteins and their functional categories in iTRAQ-based quantitative proteomic analysis. (**A**,**C**) Numbers of up- and down- accumulated proteins at 24 and 48 h, respectively; (**B**,**D**), functional categories of up- and down-accumulated proteins according to Blast2GO analysis.

An iTRAQ-based quantitative proteomic analysis was performed to investigate the proteomic responses of *C. guilliermondii* to patulin. In addition, this approach was also performed to identify the candidate proteins that are involved in patulin degradation. Using the *C. guilliermondii* protein database, a total of 928 and 1006 non-redundant proteins were identified with a global false discovery rate (FDR) below one percent in the two respective biological replicates. Among them, a total of 30 proteins (up: 9 proteins; down: 21 proteins) were differentially accumulated based upon having a fold change of >1.50 or <0.67 (patulin treatment vs. control) (Figure 4A,C).

Nine up-accumulated proteins were classified into six groups according to Blast2GO analysis (Biological process), including carbohydrate metabolic process (two proteins), cellular component organization (two proteins), energy metabolic process (one protein), oxidation-reduction process (two proteins), protein metabolic process (one protein), and response to stimulus (one protein) (Figure 4B and Table 1). Notably, the abundance of protein U7 (gi|190348612; Short-chain dehydrogenase) distinctly increased by 51 and 24 folds after co-incubation with patulin for 24 and 48 h, respectively. Down-accumulated proteins were classified into seven groups (Figure 4D and Table 2). Among the 21 down-accumulated proteins, eight proteins belong to the biological process of protein biosynthesis and modification; including components of ribosome (proteins D12–D16), elongation factor (D17), and a protein involved in biogenesis of ribosome (D18). Other proteins were involved in amino acid metabolic process, carbohydrate metabolic process, cellular component organization, energy metabolic process, lipid metabolic process, response to stimulus, and transport.

### 2.4. Changes in mRNA Abundances of Related Genes

**Figure 5:**
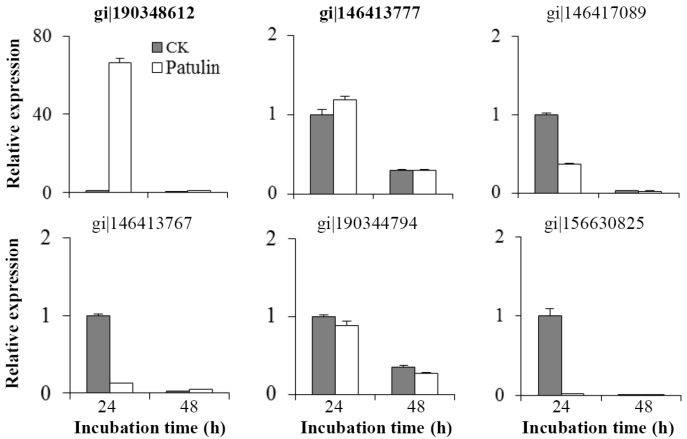
Changes in the relative expression patterns of genes encoding differential proteins. Bolded gi numbers represent up-accumulated proteins, as based upon proteomic analysis. The non-bolded gi numbers represent proteins that were down-accumulated in the proteomic analysis. Error bars indicate the standard deviations of the means from three replicates.

The abundance of mRNA corresponding to six selected differential proteins was analyzed by RT-qPCR (Figure 5). The expression patterns of four gene encoding proteins gi|190348612, gi|146417089, gi|146413767, and gi|156630825 were in accordance with their patterns at the protein level. The relative expression of the gene encoding the short-chain dehydrogenase (gi|190348612) increased by 66 folds in patulin treated *C. guilliermondii* cells at 24 h. On the other hand, no significant changes were observed in mRNA abundances of two gene encoding proteins gi|146413777 and gi|190344794. These results suggested that the differential patterns of protein abundance in response to patulin are likely regulated at the transcriptional and/or post-transcriptional levels.

## 3. Discussion

Biodegradation is a promising strategy to remove mycotoxins in food and feed. Recently, a number of microorganisms, especially yeasts, have been reported to have the ability to remove patulin [25,26]. In the present study, an antagonistic yeast *C. guilliermondii* strain 2.63 was observed to multiply to a high population in medium containing patulin at concentrations ranging from 100–500 μg/mL; however, the growth rate was inhibited at the early stage (Figure 1A). These data suggested that *C. guilliermondii* may have the ability to resist or remove patulin. Subsequently, HPLC analysis revealed that the patulin concentration was significantly reduced when it was co-incubated with *C. guilliermondii*, and the living yeast cells showed a greater efficacy for removing patulin as compared to dead cells (Figure 1B). Yeast cells remove patulin through two mechanisms: adsorption and biodegradation [26]; and the latter is dependent on yeast viability. A similar decrease in the patulin concentration was observed at six hours in both living and dead yeast treatments. It is plausible that adsorption on patulin might occur at this stage in both treatments. After that, the level of patulin exhibited a rapid reduction in treatments with living yeast cells; whereas minimal change was observed in the treatment containing dead cells. These results indicated that *C. guilliermondii* reduced ambient patulin levels primarily through a biodegradation in living cells. Furthermore, we found that intracellular proteins, rather than cell culture supernatant, demonstrated patulin degradation activity, indicating that patulin biodegradation is an intracellular event in *C. guilliermondii* (Figure 1C). These data are consistent with a previous study in *Rhodosporidium paludigenum* [27].

Prior to the application of biodegradation technology for controlling mycotoxin contamination, it is essential to identify degradation products and to also evaluate their toxicity. At the present time, two major biodegradation products of patulin have been identified as DPA and ascladiol [25]. Specifically, *R. kratochvilovae* and *R. paludigenum* are known to convert patulin to DPA [19,27], while *Saccharomyces cerevisiae* and *Kodameae ohmeri* transform patulin into ascladiol [12,28]. Ascladiol peak appears earlier than patulin peak in the HPLC-UV/DAD chromatogram, while DPA peak was eluted closely following the patulin peak [1,19,27]. In the present study, after co-incubation of patulin and *C. guilliermondii*, a new peak with a retention time of 6.0 min was eluted prior to patulin (Figure 2A), and had a maximum UV absorption of approximately 268 nm (Data not shown). Both the retention time and maximum UV absorption wavelength were consistent with E-ascladiol standard. Meanwhile, HPLC analysis did not reveal any additional new peaks after the patulin peak. Thus, we assumed that E-ascladiol was the main degradation product of patulin by *C. guilliermondii*. E-ascladiol is immediate biosynthetic precursor of patulin and has reduced toxicity compared with patulin [15]. We identified an EIC of *m*/*z* 155.0358 from biodegradation sample of patulin by LC-TOF-MS analysis, which corresponds to the molecular formula of C_7_H_8_O_4_. Although E-ascladiol and DPA have the same formula of C_7_H_8_O_4_, information gained from the retention time of EIC and the MS/MS profile confirmed that the degradation product was E-ascladiol (Figure 3). Ianiri et al. [29] reported that the production of DPA was only found in the Pucciniomycotina species of basidiomycetes, while the ascomycete *S. cerevisiae* and the bacteria *Gluconobacter oxydans* convert patulin to ascladiols. Here, we also found that *C. guilliermondii*, an ascomycetous yeast, degraded patulin into E-ascladiol instead of DPA.

Elucidation of the degradation mechanisms and identification of key functional factors in yeasts will help to enable the development of methodologies for the efficient removal of patulin in food. Ianiri et al. [29] studied the transcriptomic responses of yeast *Sporobolomyces* sp. to patulin and found that complex mechanisms were involved. In addition, they also generated a library of T-DNA insertional mutants to search genes that are functionally involved in patulin degradation [1]. The biodegradation mechanism of patulin is an enzymatic reaction. In *S. cerevisiae*, proteins involved in patulin degradation were induced by the addition of patulin. In contrast, cyclohexamide, which is a potent blocker of protein synthesis in yeast, was capable of preventing the biodegradation of patulin [30]. However, proteins that possess specific activity for the degradation of patulin have not been identified and functionally characterized until now. In the present study, the responses of *C. guilliermondii* to patulin were investigated using an iTRAQ-based quantitative proteomic analysis. A total of 30 differential proteins that are involved in 10 different biological processes were identified, and more than two-thirds of differential proteins (21 proteins) were down-accumulated in patulin treated yeast cells (Figure 4). Patulin treatment decreased the abundance of eight proteins that are involved in the processes of protein biosynthesis and modification. Additionally, proteins involved in other biological processes, such as amino acid metabolism, carbohydrate metabolism, and energy metabolism, were down-accumulated. These results indicated that patulin exposure as a stress could affect various metabolic pathways of yeast cells, which might explain the inhibitory effect of patulin on growth of *C. guilliermondii* at the early growth stage.

More attention was paid to the up-accumulated proteins that may be directly related to the resistance and degradation of patulin in yeast cells. Patulin is considered to damage cells and cellular processes via multiple electrophilic reactivity which leads to the formation of adducts with nucleophiles, such as sulfhydryl (-SH) of the antioxidant peptide glutathione (GSH) [31]. In the fission yeast *Schizosaccharomyces pombe*, patulin treatment led to GSH depletion, accumulation of reactive oxygen species (ROS), and the activation of antioxidant systems [31]. In *S. cerevisiae* and *Sporobolomyces* sp., patulin induced the up-regulation of a series of genes that are involved in oxidation-reduction process [29,32]. In the present study, the abundance of two proteins (U6 and U7) involved in oxidation-reduction process was also markedly induced by treatment with patulin (Table 1); suggesting the potential responses against oxidative stress. Notably, comparing with the control, the abundance of protein U7 (gi|190348612, short-chain dehydrogenase) in *C. guilliermondii* increased by 51 and 24 folds in the patulin treatment at 24 and 48 h, respectively. Expression of the corresponding gene was also strongly induced at 24 h (Figure 5). A decrease in the abundance of short-chain dehydrogenase at 48 h might be related to the reduction of the patulin concentration. Short-chain dehydrogenases/reductases (SDRs) constitute a large family of NAD(P)(H)-dependent oxidoreductases and have critical roles in lipid, amino acid, carbohydrate, cofactor, hormone and xenobiotic metabolisms [33]. The SDR superfamily consists of at least 140 different enzymes that are active on a wide spectrum of substrates. Ianiri et al. [29] found that genes encoding short or medium chain dehydrogenases were up-regulated under patulin stress in *Sporobolomyces* sp. It was suggested in the aforementioned study that short or medium chain dehydrogenases were potentially able to degrade xenobiotic compounds. Our study revealed additional evidence to support the hypothesis that a short-chain dehydrogenase may be directly involved in the biodegradation of patulin. Heterogeneous expression and purification of the protein are on-going, in order to verify activity of the protein in patulin degradation. Patulin as a toxic stress also induced the accumulation of a heat shock protein 70 (U9, gi|146413777). The result agreed with a recent report by Zheng et al. [34]. Heat shock protein 70 is one of the most important and conserved molecular chaperone families which protects proteins against environmental and physiologic stress [35].

## 4. Conclusions

In the present study, we found that *C. guilliermondii* could grow in the presence of various concentrations of patulin and reduce the content of patulin in culture medium. Degradation of patulin by *C. guilliermondii* was dependent on the yeast cell viability and mainly occurred inside cells. E-ascladiol was the main degradation product of patulin. An iTRAQ-based proteomic analysis identified 30 differential proteins, belonging to 10 different biological processes, that are involved in the responses of *C. guilliermondii* to patulin. Among them, nine proteins were up-accumulated and 21 proteins were down-accumulated in patulin treated yeast cells. Notably, a short-chain dehydrogenase was significantly induced by patulin at both the protein and mRNA levels, suggesting its importance to enable the biodegradation of patulin in *C. guilliermondii*. The findings presented in this study will serve as a foundation for the development of novel technologies to control patulin contamination. In the future, we envision that commercially formulated patulin-degrading enzymes can be cost effectively produced and applied in fruit-derived products in an effort to remove patulin.

## 5. Materials and Methods

### 5.1. Yeast Strain

The *Candida guilliermondii* strain 2.63 was obtained from the Institute of Microbiology, Chinese Academy of Sciences, Beijing. The strain was preserved in glycerol and stored at −80 °C. According to the description by Fan et al. [22], the strain was inoculated and maintained in nutrient yeast dextrose broth (NYDB, 1 g of beef extract, 5 g of soybean peptone, 5 g of NaCl, 7 g of yeast extract, and 10 g of glucose within 1000 mL distilled water) medium at 28 °C prior to the experiment.

### 5.2. Effect of Patulin on the Growth of *C. guilliermondii*

The assay was performed according to the method of Dong et al. [12] with some modifications. Yeast cells of *C. guilliermondii* were cultured in NYDB at 28 °C on a rotary shaker at 200 rpm, and collected at the late log phase by centrifugation at 8000× *g* for 3 min. The harvested yeast cells were resuspended at a concentration of 1 × 10^5^ cells/mL in NYDB (pH 5.0) with patulin (Sigma-Aldrich, St. Louis, MO, USA) ranging from 0, 100, 250, and 500 μg/mL. The yeast was cultured at 28 °C and 200 rpm, and sampled every 24 h. The cell concentration of each sample was counted using a hemacytometer. There were three replicates per treatment and each experiment was repeated twice.

### 5.3. Biodegradation Assay of Patulin

A biodegradation assay was carried out according to the method of Dong et al. [12] and Zhu et al. [27] with some modifications. Yeast cells cultured in NYDB for 48 h were collected by centrifugation at 8000× *g* for 3 min. After washing three times using sterile distilled water, the yeast cells were divided into two groups. Yeast cells in group I were resuspended in 50 mM MES buffer (pH 5.0) containing 50 μg/mL patulin at a concentration of 1 × 10^9^ cells/mL. Yeast cells in group II were initially boiled for 20 min in order to kill all cells. The dead cells were then suspended in 50 mM MES buffer (pH 5.0) containing 50 μg/mL patulin at a concentration of 1 × 10^9^ cells/mL. The MES buffer (pH 5.0) containing 50 μg/mL patulin without yeast cells was used as control. Aliquots of 2 mL above mixtures were added into 10 mL tubes, cultured at 28 °C (200 rpm), and sampled at 6, 24 and 48 h. The sampled cultures were centrifuged at 8000× *g* for 5 min. Then, the supernatants were filtered through a 0.22 μm filter for patulin detection. There were three replicates per treatment and each experiment was repeated twice.

In order to determine the localization of patulin biodegradation, yeast cells and culture supernatant were separated after 48 h of incubation in NYDB by centrifugation at 8000× *g* for 10 min. Sample I: Aliquot of 2 mL culture supernatant was mixed with 0.5 mL of 250 mM MES buffer (pH 5.0) and filtered through a 0.22 μm filter. Sample II: yeast cells (1 × 10^9^ cells) were suspended with 2 mL 50 mM MES buffer (pH 5.0), and broken with glass beads (Sigma-G8772) by vortexing (10 times, 1 min for each time). Intracellular protein extracts were collected by centrifugation at 15,000× *g* for 30 min at 4 °C. Aliquots of 2 mL Sample I and Sample II were co-incubated with patulin at a final concentration of 50 μg/mL, respectively. The MES buffer (pH 5.0) containing 50 μg/mL patulin was used as a control. The mixtures were incubated at 28 °C (200 rpm), and sampled at 24 h for patulin detection. There were three replicates per treatment and each experiment was repeated twice.

### 5.4. HPLC Analysis for Patulin and Biodegradation Products

Patulin and biodegradation products were determined by high-performance liquid chromatography (HPLC) according the methods described by Zong et al. [36]. For determination of patulin degradation products, a biodegradation assay was performed according to the above methods. Yeast cells at a concentration of 1 × 10^9^ cells/mL were co-incubated with 50 μg/mL patulin in MES buffer (pH 5.0), and sampled at 2, 12, 24 and 48 h. HPLC analysis was performed by injecting 10 μL of the filtrate extract into a liquid chromatography (Waters Corp., Milford, MA, USA) equipped with an auto sampler (Waters 2498), binary HPLC pump (Waters 1525), and an UV/Visible Detector (Waters 2487). A C18 column (5 μm, 250 × 4.6 mm, Intersil ODS-3, GL Sciences, Tokyo, Japan) was used and the column oven was set at 25 °C. The mobile phase consisted of a mixture of water and acetonitrile (90:10, *v*/*v*) at a flow rate of 1 mL/min in the isocratic elution mode and the detection wavelength was 276 nm. For the identification biodegradation products, a standard of E-ascladiol (prepared in our lab) was used.

### 5.5. LC-TOF/MS and LC-TOF-MS/MS Analysis for Biodegradation Products

The assay was carried out according to the method of Dong et al. [12] with slight modifications. The identification and confirmation of the major degradation product of patulin was performed by an Agilent 1290 Series containing a degasser, quaternary pump, column oven and automatic sampler, and interfaced with an Agilent 6540 Q-TOF accurate mass spectrometer with an electrospray ionization source. Samples were analyzed in the negative ionisation mode. An Agilent Zorbax SB-C18 column (100 × 2.1 mm, 1.8 μm) was used and the mobile phase was a mixture of water and acetonitrile (90:10, *v*/*v*) with a flow rate of 0.3 mL/min. The mass spectrometer conditions were as follows: gas temperature, 325 °C; drying gas (nitrogen), 12 L/min; nebuliser pressure, 40 psi; capillary voltage, 3500 V; skimmer, 65 V; and octopole radiofrequency voltage, 750 V. The mass scan was over the range of *m*/*z* 50–1000. Data analysis was performed with Agilent Mass Hunter Workstation software. For MS/MS analysis, collision-induced dissociation (CID) of 10 eV was used to obtain a distinct fragmentation.

### 5.6. Protein Extraction

Yeast cells were co-incubated with patulin at a concentration of 50 μg/mL and collected after culture period of 24 and 48 h. Approximately 5 × 10^8^ cells were harvested by centrifugation at 10,000× *g* for 10 min at 4 °C. After the removal of supernatant, the cell suspension was washed with PBS, transferred to a 1.5 mL microcentrifuge tube and was re-centrifuged under the same conditions. The cell pellet was suspended in 300 μL of extraction buffer (0.5 M TEAB, 7 M Urea, 2 M Thiourea, 20 mM MgCl_2_, 10 mM Tris-(2-carboxyethyl) phosphine (TCEP) and 1 mM PMSF) and was broken by vortexing (10 times, 1 min for each time) in the presence of glass beads. Then, the samples were centrifuged at 15,000× *g* for 30 min at 4 °C to remove cell debris, and the supernatant was collected. The concentration of protein was measured using the Bradford method.

### 5.7. Protein Digestion and iTRAQ Labeling

The procedures of protein digestion were modified from the previously described method of Liu et al. [37]. A total of 50 μg proteins from each sample was reduced with 1 mM TCEP for 4 h at 37 °C, and alkylated with 1 mM methylmethanethiosulfonate (MMTS) for 1 h at room temperature. Samples were then digested with sequencing grade modified trypsin (Promega, Madison, WI, USA) for 16 h at 37 °C at a 1:50 trypsin-to-protein mass ratio. The resulting tryptic peptides were vacuum concentrated and re-suspended in 30 μL of iTRAQ dissolution buffer and labeled using an iTRAQ Reagents 4-plex Kit (Applied Biosystems, Framingham, MA, USA) according to the manufacturer’s instructions.

Each iTRAQ™ 4-plex reagent vial (114 to 117) was resuspended in 70 μL of ethanol and incubated at room temperature for 1 h with the respective peptide sample, as follows: 114-CK-24 h (peptides from *C. guilliermondii* un-treated with patulin for 24 h); 115-Patulin-24 h (*C. guilliermondii* treated with patulin for 24 h); 116-CK-48 h (*C. guilliermondii* un-treated with patulin for 48 h); 117-Patulin-48 h (from *C. guilliermondii* treated with patulin for 48 h). After 2 h of incubation at room temperature, the labeling reaction was quenched by adding 100 μL of ultrapure water to each vial. Samples were incubated at room temperature for 1 h. Subsequently, the peptide mixtures were pooled, desalted using a ZipTip C18 micropipette tip (EMD Millipore, Billerica, MA, USA), dried with a SpeedVac (Thermo Fisher, San Jose, CA, USA), and stored at −80 °C prior to LCMS/MS analysis. Two independent biological replicates were processed and analyzed.

### 5.8. NanoLC-MS/MS and Bioinformatic Analysis

Peptides were analyzed in a Triple TOF 5600+ mass spectrometer (AB SCIEX, Foster City, CA, USA), coupled with a NanoLC system (NanoLC-2D Ultra Plus, Eksigent, Dublin, CA, USA) following the method described by Liu et al. [37] with some modifications. Peptides were dissolved in 100 μL solvent A (0.1% TFA in 2% ACN). Ten μL of each sample was loaded into a C18 trap column (100 μm × 20 mm) and subsequently eluted from the trap column over the C18 analytic column (75 μm × 150 mm) at a flow rate of 300 nL/min in a 90 min linear gradient ranging from 5% to 30% mobile phase B (0.1% FA in 98% ACN). The information dependent acquisition (IDA) mode was used to acquire MS/MS data. Precursor ions were selected across the mass range of 350–1500 *m*/*z* using a 250-ms accumulation time per spectrum. Tandem mass spectra were recorded in high sensitivity mode (resolution > 15,000) with both the rolling collision energy and iTRAQ reagent collision energy adjustment on.

Protein identification and quantification were performed with ProteinPilot™ Software 4.5 (AB SCIEX, Foster City, CA, USA), and database searches were carried out using the *C. guilliermondii* protein database (NCBI). The utilized search parameters used were as follows: (1) Sample Type: iTRAQ 4-plex (Peptide Labeled); (2) Cysteine Alkylation: MMTS; (3) Digestion: Trypsin; (4) Instrument: Triple TOF 5600; (5) ID Focus: Biological modifications; (6) Quantitate: Yes; (7) Bias Correction: Yes; (8) Background Correction: Yes; (9) Search effort: Thorough; (10) FDR Analysis: Yes. For iTRAQ quantification, the peptide for quantification was automatically selected by the Pro Group algorithm to calculate the reporter peak area, error factor (EF) and the *p*-value. A reverse database search strategy was adopted to estimate the false discovery rate (FDR) for peptide identification and FDR were all set as lower than 1% at the protein level. Proteins with fold change >1.5 or <0.67 (*p*-value < 0.05) between any two samples were considered to be differentially expressed proteins. The final fold change was calculated as the average value obtained from two replicates.

Blast2GO Software was used for functional analysis of the identified proteins (Bioinformatics Department, CIPF, Valencia, Spain,) [38]. Proteins were categorized according to biological process within their GO annotation [39].

### 5.9. RNA Isolation and Reverse Transcription-Quantitative PCR (RT-qPCR)

Total RNA was isolated from 5 × 10^8^ yeast cells of each sample using TRIzol (Tiangen Biotech, Beijing, China). Genomic DNA elimination and first-strand cDNAs generation were performed using PrimeScript RT reagent Kit with gDNA Eraser (Perfect Real Time, TaKaRa Code. DRR047, Dalian, China). The RT-qPCR reactions were carried out in a total volume of 20 μL, containing 10 μL of SYBR Premix Ex Taq^TM^ (TaKaRa Code. RR820), 0.4 μL of each primer (10 μM), 0.4 μL of ROX Reference Dye, 2 μL of cDNA, and 6.8 μL of RNase-free water. An ABI Step One Plus Real-Time PCR Systems (Applied Biosystems, Foster City, CA, USA) was used. The primers used for amplification were designed using Primer Express software 3.0 (Applied Biosystems), and the sequences were listed in Table 3. Transcript levels were normalized against the 5.8S rRNA (GenBank accession number AB568347.1) according to Li et al. [40] and relative expression levels were calculated using the 2^−ΔΔCT^ method [41].

### 5.10. Statistical Analysis

Statistical analyses were performed with SPSS version 11.5 (SPSS Inc., Chicago, IL, USA) and analyzed by a one-way analysis of variance (ANOVA). Mean separations were performed by Duncan’s multiple range tests. Differences at *p* ≤ 0.05 were considered significant.

## Figures and Tables

**Table 1:** Identification of differentially up-accumulated proteins with an iTRAQ-based quantitative proteomic analysis of *C. guilliermondii* that was treated with or without patulin.

Protein ID	GI Number	Protein Name	Fold Change (Patulin/Control)	
			24 h	48 h
**Carbohydrate metabolic process**				
U1	gi|190345021	Glucose-6-phosphate isomerase	1.39	1.56
U2	gi|190348104	Glyceraldehyde 3-phosphate dehydrogenase	2.97	4.09
**Cellular component organization**				
U3	gi|146411959	NADPH-dependent methylglyoxal reductase	6.70	4.33
U4	gi|146420860	NADPH-dependent methylglyoxal reductase	1.89	1.64
**Energy metabolic process**				
U5	gi|146416915	Phosphoglycerate kinase	1.10	1.83
**Oxidation-reduction process**				
U6	gi|146414467	Potential oxidoreductase	6.83	9.54
U7	gi|190348612	Short-chain dehydrogenase	51.1	24.0
**Protein metabolic process**				
U8	gi|190347960	Cell division control protein 48	1.51	1.61
**Response to stimulus**				
U9	gi|146413777	Heat shock protein 70	1.42	1.63

**Table 2:** Identification of differentially down-accumulated proteins with an iTRAQ-based quantitative proteomic analysis of *C. guilliermondii* treated with or without patulin.

Protein ID	GI Number	Protein Name	Fold Change (Patulin/Control)	
			24 h	48 h
**Amino acid metabolic process**				
D1	gi|146417089	NADP-specific glutamate dehydrogenase	0.52	0.45
D2	gi|146417813	Serine hydroxymethyltransferase	0.24	0.65
**Carbohydrate metabolic process**				
D3	gi|146413757	Isocitrate lyase	0.60	0.57
D4	gi|146414542	Mannose-1-phosphate guanyltransferase	0.63	0.49
D5	gi|146415901	S-adenosylmethionine synthase	0.54	0.53
**Cellular component organization**				
D6	gi|146413767	Histone H4	0.50	0.38
**Energy metabolic process**				
D7	gi|190345530	Ketol-acid mitochondrial	0.13	0.63
**Lipid metabolic process**				
D8	gi|190344794	Acyl-Coenzyme A oxidase 4	0.24	0.46
D9	gi|146416129	Cytochrome P450 (Lanosterol 14-alpha demethylase)	0.39	0.60
D10	gi|190346072	Fatty acid synthase subunit beta	0.39	0.74
**Protein biosynthesis and modification**				
D11	gi|146423689	3-isopropylmalate dehydratase	0.66	0.58
D12	gi|269969601	40S ribosomal protein s1	0.38	0.66
D13	gi|146415016	40S ribosomal protein S19-B	0.08	0.91
D14	gi|146419667	60S ribosomal protein L17-B	0.60	0.95
D15	gi|146418206	60S ribosomal protein L30	0.28	0.45
D16	gi|146417697	60S ribosomal protein L4-b	0.10	0.57
D17	gi|152032427	Elongation factor 1-alpha	0.48	0.31
D18	gi|190346643	Nucleolar protein 56	0.90	0.58
**Response to stimulus**				
D19	gi|146420955	5-methyltetrahydropteroyltriglutamate homocysteine methyltransferase	0.37	0.60
D20	gi|156630825	Histone H3.1/H3.2	0.46	0.48
**Transport**				
D21	gi|146412540	ABC transporter ATP-binding protein ARB1	0.98	0.48

**Table 3:** Primers used for RT-qPCR.

Gene	Primer Sequences (5′–3′)
*5.8S rRNA*	F: GCTGTCGACCTCTCAATGTATTAGG
	R: TAAGGCCGGGCCAACAAT
*gi|190348612*	F: TCTTTCGGCCCACTTTTGAA
	R: AAGCGAATGGGTCCCAAGAC
*gi|146413777*	F: CCACCAACCAAAGAGCATTGA
	R: GGTTTGAGCAGAAGAAGACAAGGT
*gi|146417089*	F: AAAGGCCACTGGTGGAAAGG
	R: CAGCGTATTGGGCAACGTTA
*gi|146413767*	F: ATTAGGAAAGGGAGGCGCTAA
	R: TTCTAATAGCTGGCTTGGTGATACC
*gi|190344794*	F: CGAAGCCACCGAGGAGTTT
	R: AAGAATGAGCAGCACCACCAA
*gi|156630825*	F: CAAGAAAGTCCACTGGTGGTAAGG
	R: TCCAGTAGAAGGAGCGGATTTT
